# Characteristics and quality of life of patients presenting to cancer support centres: patient rated outcomes and use of complementary therapies

**DOI:** 10.1186/1472-6882-13-169

**Published:** 2013-07-11

**Authors:** Bonnie J Furzer, Kemi E Wright, Anna S Petterson, Karen E Wallman, Timothy R Ackland, David JL Joske

**Affiliations:** 1 The University of Western Australia, M408, 35 Stirling Hwy, Crawley, WA 6009, Australia; 2 SolarisCare Foundation Collaborative Research Team, PO Box 7144, Shenton Park, WA 6008, Australia; 3 Sir Charles Gairdner Hospital, Haematology Care Center, Ground Floor, E Block, Hospital Ave, Nedlands, WA 6009, Australia; 4 Edith Cowan University, 270 Joondalup Drive, Joondalup, WA 6027, Australia

**Keywords:** Cancer patients, Complementary therapies, Quality of life, Supportive care, Symptom distress

## Abstract

**Background:**

In order to effectively target and provide individualised patient support strategies it is crucial to have a comprehensive picture of those presenting for services. The purpose of this study was to determine the characteristics and patient rated outcomes of individuals presenting to SolarisCare cancer support centres and their choices regarding complementary and integrated therapies (CIT).

**Methods:**

A cohort with a current or previous cancer diagnosis aged 18 – 87 years presenting to a SolarisCare centre during a 5-day period completed a questionnaire. Four SolarisCare centres participated in the trial including regional and metropolitan locations. Outcomes included medical and demographic characteristics, CIT variables and patient rated outcomes (PROs) including quality of life (QoL).

**Results:**

Of the 95 participants (70.3%) who completed the survey, the mean age was 60.5 years with 62% currently receiving treatment. Eighty percent of the sample had at least one other comorbid condition, with the most popular CIT being relaxation massage. Of the PROs, QoL was significantly lower than norms for the Australian population and other mixed cancer populations. No notable differences were seen between genders, however significantly poorer outcomes were found for the younger age group. Fifty percent of the population did not meet physical activity recommendations, and musculoskeletal symptoms explained between 25-27% of variance in QoL.

**Conclusions:**

A greater understanding of the health profiles of patients presenting to supportive care centres and their use of CIT, provides Western Australian health professionals with key information to ensure the safety of supportive care practices, as well as fosters optimal patient outcomes and enhances the integration of supportive care strategies within mainstream medical care.

## Background

Cancer is one of the largest contributors to mortality and morbidity in Australia accounting for 19% of the total burden of disease and 12.5% of deaths worldwide [1]. There were 108,363 new cases of cancer diagnosed in Australia in 2007, with the lifetime risk of diagnosis below the age of 85 at 1 in 2 [1]. Concurrently, advances in early diagnosis and treatment have seen increases in survival rates with cancer patients accounting for approximately 3.2% of the Australian population, with a predicted growth rate of 2% annually [2]. Support services have emerged as an integral aspect of patient management during and post treatment, with interventions ranging from pharmacological to lifestyle modification, and branching out to complementary integrated therapies (CIT). With 63% of cancer patients’ alive 5 years post treatment, targeted services are crucial to help manage long-term medical and psychosocial health, including quality of life (QoL), fatigue and pain [1].

Cancer is most prevalent in Australians over 65 years of age, affecting 11% of males and 4% of females [3]. The combination of age related decline, comorbidites and the side effects of multiple treatments creates a population with unique characteristics and specific health needs. While fear of recurrence and development of secondary cancers are the biggest concerns for patients one year post diagnosis, a study by Baker and colleagues found 67.1% of patients were also concerned about a physical health problem, such as fatigue and loss of strength [4]. Research has consistently shown that cancer patients have low or reduced levels of QoL from initial diagnosis often for several years post-treatment, in addition to significant psychological distress commonly manifested as depression and/or anxiety [5]. Also occurring during treatment and often persisting 5–10 years post treatment is cancer related fatigue (CRF), considered one of the most debilitating symptoms, affecting 70-100% of patients [6]. In addition to CRF, musculoskeletal symptoms have been widely documented in both patients and survivors [7], with strong associations between symptoms such as joint and muscle pain, and weakness and fatigue [8].

A study of supportive care strategies across six countries, including Australia, reported 35-60% of adults have used some type of non-conventional treatment or therapy [9]. In Australia, the use and prevalence of CIT is increasing with an estimated $2.3 billion spent in 2000 [10]. In 1996, CIT use amongst a cancer population was reported to be 21.9%, with 75% having tried more than one therapy and remarkably 40% not discussing usage with their physician [11]. The most common motivations for using CIT are to relieve symptoms/side effects, assist in disease management, improve immunity, improve QoL and increase sense of control [12,13].

With the increased utilisation of support strategies including CIT, there is a crucial need to identify the characteristics of those presenting to a cancer support centre. SolarisCare is a unique cancer support organisation in Australia, offering drop-in centres, mostly within hospital settings, that provide a quiet area, access to information, and a range of CIT for cancer patients and carers. These sessions are carefully supervised and therapists are selected to ensure patients’ physical and psychological safety; no adverse medical events have occurred in 11 years’ of practice.

An increased understanding of cancer patients currently accessing CIT and supportive care services enables the refinement of patient needs and allows the tailoring and specificity of services to achieve optimal patient care and outcomes. Therefore, the aim of this study was to determine medical and demographic characteristics and patient rated outcomes (PROs) of individuals receiving CIT at SolarisCare cancer support centres in Western Australia.

## Methods

The study was conducted over a five-day period at four SolarisCare cancer support centres spread throughout the South West and Greater Southern regions of Western Australia. The data from this study forms part of a broader study, which was granted ethics approval by The University of Western Australia’s Human Research Ethics Committee (RA/4/1/2113) and the Human Ethics & Research Committee at Sir Charles Gairdner Hospital (Ref. 2009–61). Written informed consent was obtained from all participants and eligibility requirements for the study included the ability to provide informed consent; a diagnosis of cancer; and presentation to a SolarisCare centre with the intent to receive services.

Demographic and medical questions were designed based on previous research and patient information sheets currently in use at SolarisCare (Additional file 1). Additionally, participants completed a questionnaire package that assessed quality of life using the Functional Assessment for Cancer Therapy (FACT-G) [14] and the Short Form 36 Health Survey (SF-36) [15]. Fatigue was measured using the Schwartz Cancer Fatigue Scale (SCFS) [16]; musculoskeletal symptoms were measured with the Muscle Joint Measure (MJM) [8]; and physical activity was measured using the International Physical Activity Questionnaire (IPAQ) [17]. The reliability and validity of all the scales used has previously been established, with these scales widely utilised in cancer research.

### Statistical analysis

All analyses were conducted using SPSS® software, version 19. Outcome variables were categorised and analysed, and cross tabulations of CIT by demographic and clinical variables were constructed. Preliminary descriptives were performed on all PROs to ensure no violation of the assumptions of normality, linearity and homoscedasticity, with independent sample *t*-tests used to assess differences between groups and Pearson correlations used to explore the relationship between variables.

## Results

### Participant characteristics and CIT use

**Figure 1:**
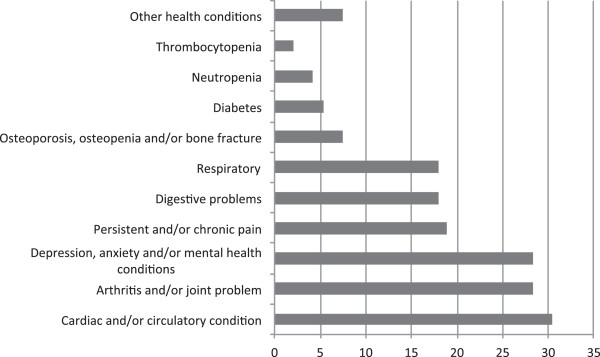
**Percentage of comorbidities of study participants (n = 95).** % of sample.

Of the 135 eligible participants, 95 (70.3%) agreed to participate, with 46% (n = 44) recruited from Sir Charles Gardiner Hospital, 25% (n = 24) from St John of God, 18.9% (n = 18) from Bunbury and 9.5% (n = 9) from Albany. The patients’ characteristics and medical information are provided in Table 1. Forty three percent of the population had metastases. The most comon sites were bone (37.5%; n = 15), liver (35%; n = 14) and lung (27.5%; n = 11). Of the participants, 62% (n = 59) were currently receiving treatment with 66% (n = 39) receiving radiotherapy, 32% (n = 19) chemotherapy, and 22% (n = 13) hormone therapy. Additionally, 70% (n = 63) had undergone a cancer related surgery. Forty percent of participants (n = 38) reported one other comorbidity, 24.2% (n = 23) had 2–3, 15.8% (n = 15) had greater than 4, with only 20% (n = 19) presenting with none. The types of comorbidities are shown in Figure 1.

**Table 1:** Demographic and medical characteristics of study participants

**Variable**	**n = 95**	**Variable**	**n = 95**
*Demographic profile*		*Medical profile*	
Gender		Cancer site	
Male	33 (34.7%)	Breast	22 (23.2%)
Female	62 (65.2%)	Gynaecological	11 (11.6%)
Age (±SD; range)	60.49 (12.65; 28–87)	Colorectal	9 (9.5%)
25-34	1 (1.1%)	Prostate	8 (8.4%)
35-44	10 (10.5%)	Kidney	4 (4.2%)
45-54	23 (24.2%)	Lung	6 (6.3%)
55-64	20 (21.1%)	Haematological	7 (7.4%)
65-74	28 (29.5%)	Head and neck	6 (6.5%)
75+	13 (13.7%)	Brain	6 (6.3%)
Employment		Melanomas	4 (4.2%)
Not employed	13 (13.7%)	Pancreatic	3 (3.2%)
Casual	5 (5.3%)	Bone	3 (3.2%)
Part time	10 (10.5%)	Stomach	1 (1.1%)
Full time	3 (3.2%)	Don’t know	5 (5.3%)
Sick Leave	19 (20.0%)	Allied health professionals consulted	
*Months on sick leave* (±SD; range)	6.55 (4.17; 0.5-16)	Psychologist/counsellors	23 (24.2%)
Retired	45 (47.4%)	Physiotherapy/chiropractic	13 (13.7%)
*Retired since diagnosis*	19 (20%)	Yoga/pilates/exercise	11 (11.6%)
Physical activity level	*n = 92*	Medical specialist	11 (11.6%)
Low	48 (52.2%)	Support group	10 (10.5%)
Moderate	36 (39.1%)	Naturopath/herbalist	10 (10.5%)
High	8 (8.7%)	Dietician/nutritionist	8 (8.4%)
Days of activity		Supplement use	62 (65.9%)
< 5 days a week	46 (50%)	Self-selected	27 (28.4%)
≥ 5 days a week	46 (50%)	Prescribed	23 (24.2%)
		Self-selected and prescribed	12 (12.6%)
		Don’t know	1 (1.1%)

**Figure 2:**
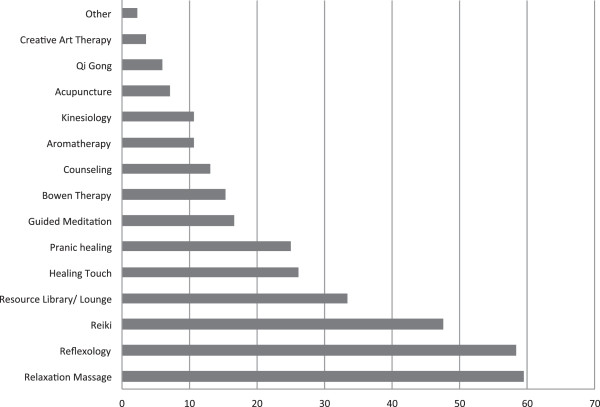
**Frequency of complementary therapy use (n = 95).** % usage.

The total number of CIT sessions attended by participants was variable with 43.2% (n = 41) attending less than 6 sessions, 21.1% (n = 20) between 6 and 12 sessions, 25% (n = 24) greater than 12 and 10.5% (n = 10) not providing data. Additionally, participants recorded the number of different therapies utilised, with 31.6% (n = 30) trying 1–2 therapies, 31.6% (n = 30) 3–4 therapies and 25.4% (n = 24) greater than five therapies. The types of CIT used by participants are shown in Figure 2.

### Patient rated outcomes

There were statistically no significant differences in FACT-G (72.9 ± 17.7, t = −0.293, p = 0.770) and SCFS (15.1 ± 6.1, t = −1.498, p = 0.138) scores between the population under study and those of other mixed cancer. However, the MJM and SF-36 scores, when compared to Australian based norms [15] or other mixed cancer populations [8,18,19] showed a number of statistically significant differences illustrated in Table 2. Differences were evident in the range and median ages of the SF-36 mixed cancer sample (median age = 44.2, range 18–77, female 73%, male 27%), which may account for some of the variation.

**Table 2:** Patient rated outcomes compared with mixed cancer and Australian population norms

	**Snapshot study**		**Means of other cancer pop.**				**AUS. ABS. general population**		
	**n**	**Mean ± SD**	**n**	**Test value**	**t**	**p**	**Test value**	**t**	**p**
**Physical component score**	95	40.90 ±10.60	1183	45.15	−3.91	<0.001**	49.70	−8.09	<0.001**
**Physical functioning**	95	60.67 ±24.96	1183	77.27	−6.48	<0.001**	82.5	−8.53	<0.001**
**Role physical**	95	45.42 ±28.15	1183	45.42	-.001	.999	79.8	−11.90	<0.001**
**Vitality**	95	43.22 ±21.76	1183	55.53	−5.51	<0.001**	64.5	−9.53	<0.001**
**General health**	95	42.68 ±12.04	1183	61.38	−2.28	0.025*	71.6	−6.22	<0.001**
**Mental component score**	95	42.00 ±12.48	1183	49.94	−6.20	<0.001**	50.10	−6.38	<0.001**
**Bodily pain**	95	60.94 ±28.05	1183	65.99	−1.75	0.82	76.8	−5.51	<0.001**
**Social functioning**	95	57.10 ±28.63	1183	76.08	−6.46	<0.001**	84.9	−9.46	<0.001**
**Role emotional**	95	61.05 ±28.77	1183	70.58	−3.23	0.002*	82.8	−7.37	<0.001**
**Mental health**	95	66.26 ±21.28	1183	73.04	−3.10	0.003*	75.9	−4.41	<0.001**
**Muscle joint Measure**	91	1.19 ±0.85	317	0.94	2.78	0.007*			
**Cramps**	91	0.97 ±0.91	317	0.93	.472	.638			
**Weakness**	91	1.27 ±1.09	317	0.81	3.98	<0.001**			
**Myalgias**	91	1.25 ±1.17	317	1.10	1.21	.229			
**Arthralgias**	91	1.27 ±1.18	317	0.98	2.36	.020*			

Results are mean ± SD (n = 95).* p < .05, **p < .001 2-tailed.

Analyses of scores recorded on the SF-36 showed no significant differences between male and females in the Physical Component Score (PCS; Male 41.14 ± 11.20; Female 40.78 ±10.34; t = 0.377, *p* = 0.88) or in the Mental Component Score (MCS; Male 43.34 ± 10.43; Female 41.35 ± 13.39; t = 0.237, *p* = 0.47). However, scores on the General Health (GH) subscale were significantly lower for males compared to females (Male 38.88 ± 11.86; Female 44.52 ± 11.78; t = −2.183, *p* = 0.03). Results showed the MCS was significantly lower for those ≤ 62 years (39.67 ± 12.13) compared to those aged ≥ 63 years (44.58 ± 12.48; t = −1.943, p = 0.05). In addition, there was a significant difference for Social Functioning (SF; Age ≤ 62 n = 34.81, SD = 12.17; Age ≥ 63 n = 41.82, SD = 11.92; t = −2.828, *p* = 0.006) with lower scores for the younger age group. When exploring the FACT-G and its subscales, social wellbeing (SWB), functional wellbeing (FWB) and total FACT-G scores were all significantly lower for those ≤ 62 years, shown in Table 3.

**Table 3:** Examination of FACT-G and subscales by population age categories (n = 95)

	**Age ≤ 62**			**Age ≥ 63**				
	**n**	**Mean**	**SD**	**N**	**Mean**	**SD**	**t**	**p**
**Physical wellbeing**	50	17.43	6.26	45	18.65	7.22	0.148	0.38
**Social wellbeing**	50	19.73	6.36	45	22.35	5.96	−2.065	0.042*
**Emotional wellbeing**	50	16.86	4.12	45	17.32	5.93	0.730	0.66
**Functional wellbeing**	50	15.20	6.63	45	18.70	6.52	−2.950	0.011*
**FACT-G**	50	69.22	17.34	44	77.18	17.27	−2.227	0.028*

*p < 0.05.

Inter-correlations between the MJM total score and subscales were medium to large in sizes, with all values for *r* ≥ 0.30 (Table 4). A strong negative relationship was shown between the PCS as measured by the SF-36, and musculoskeletal symptoms measured by the MJM (r = −0.501, n = 92, *p* = <.001), with high levels of symptoms associated with lower levels of physical functioning. Independently, 3 of the 4 subscales of the MJM showed moderate to strong negative relationships with PCS and its subscales (Table 5). Additionally, there was a strong negative correlation between Physical Wellbeing (PWB) measured by the FACT-G and musculoskeletal symptoms, including weakness (r = −0.548, n = 91, *p* < 0.001), myalgias (r = −0.518, n = 91, *p* < 0.001), and arthralgias (r = −0.398, n = 91, *p* < 0.001), as measured by the MJM (r = −0.521, n = 92, *p* < 0.000).

**Table 4:** Inter-correlations within the muscle and joint measure (MJM) (n = 95)

**Measure**	Muscle joint measure total score &subscales*			
	**Total**	**Arthralgias**	**Myalgias**	**Cramps**
**Arthralgias**	.765	-		
**Myalgias**	.860	.544	-	
**Cramps**	.669	.331	.424	-
**Weakness**	.834	.466	.688	.453

*All *p* ≤ .001.

**Table 5:** Pearson correlations between measures of physical functioning through patient rated outcomes and musculoskeletal symptoms (n = 95)

	**Muscle joint measure total scores and subscales**				
	**Cramps**	**Arthralgias**	**Myalgias**	**Weakness**	**Total score**
**SF-36:**					
**General health**	-.159	-.305**	-.282**	-.393**	-.318**
**Bodily pain**	-.258*	-.517**	-.448**	-.359**	-.451**
**Role physical**	-.179	-.357**	-.467**	-.444**	-.422**
**Physical functioning**	-.232*	-.513**	-.445**	-.455**	-.490**
**Vitality**	-.044	-.194	-.250**	-.344**	-.273**
**Physical component Score**	-.235*	-.511**	-.488**	-.463**	-.501**
**Mental component score**	-.080	-.118	-.158	-.241*	-.192
**Schwartz cancer fatigue scale**	.246*	.054	.024	.091	.121
**FACT-G:**	-.205	-.341**	-.410**	-.405**	-.440**
**Physical wellbeing**	-.324**	-.398**	-.518**	-.548**	-.574**
**Functional wellbeing**	-.124	-.229*	-.319**	-.318**	-.321**
**Social wellbeing**	-.092	-.079	-.173	-.121	-.149
**Emotional wellbeing**	-.026	-.292**	-.137	-.148	-.200

*p < .005, **p < .001 2-tailed.

## Discussion

With the two major SolarisCare centres being located within treating hospitals it is not surprising that 62% of the study population were currently receiving treatment. This highlights the crucial need for continual communication with doctors and specialists. Seventy percent of patients reported using supplements, with a large portion self-selecting their supplements. This raises considerable concerns about the potential for toxic interactions with conventional treatments and the potential lack of disclosure to doctors. Communication between patient and doctors on CIT use had been shown to be poor [12] with lack of education of both nurses and doctors [13], as well as a lack of protocols and guidelines being the biggest barriers to communication.

This study had a higher percentage of women (65.2%) compared to men (34.7%), which may suggest that SolarisCare centres were not considered as appealing by male cancer patients or may reflect the large breast cancer practice at one hospital in particular. With equally high rates of cancer in males, it is important for support services to be directed towards men and the medical professionals involved in their treatment. This should be considered in future research and service provision.

As shown in previous research, the cancer population described in this study exhibited significantly lower scores on PROs compared to the general population [5,15], additionally a number of variables were also significantly lower than in a mixed cancer population [8,14,18,19]. It is notable that only one variable, GH, showed a statistically significant difference between males and females. However, due to the greater proportion of women this study was not adequately powered to comprehensively examine gender differences and future reseach should investigate the impact of gender further.

Investigation of the impact of age demonstrated those in the younger cohort (≤ 62) were found to have significantly worse outcomes than older patients. This is in accord with research by Baker and colleagues (2005) who found more problems reported by younger survivors (18–54 years) [4]. Researchers have previously attributed the discrepancies between younger and older cancer patients to a number of factors including increased demand on younger patients in terms of social and economic variables, and a difference in the perception or expectation of health and QoL as reflected in subjective assessment [4,20]. Future research should investigate this relationship further through the utilisation of subjective and objective measures which provide age matched normative values.

It is encouraging that the data suggests those who are in need of support services are accessing the resources available at SolarisCare whether through recommendation by health professionals, other patients or self-selected. Research has highlighted the long-term nature of a number of symptoms associated with cancer treatment, making it important to address the ongoing health of those patients and survivors who may exhibit better physical coping in the short term. Locality, lack of information and accessibility could be barriers for patients accessing support services when they are no longer receiving treatment or are mulitple years post treatment.

In this population, musculoskeletal symptoms helped to explain 25% of the variance in respondents’ score on the PCS (*r* = −0.501) and 27% of the score on the PWB scale (*r* = −0.521) of the FACT-G. This demonstrates the importance of addressing symptom distress as part of ongoing patient care. Whether the musculoskeletal symptoms are a result of the cancer itself, treatments, or comorbidities such as arthritis or age-related decline, long-term management strategies in addition to acute use of CIT need to be encouraged.

Physical activity is a lifestyle intervention that research has shown to be safe and effective for alleviating many of the negative side effects of cancer and its treatments [21], however 50% of this population had low activity levels and did not meet current health recommendations. Research by Blanchard and colleagues (2004) found that cancer survivors who met more than one lifestyle recommendation reported better QoL than those who met only one [22]. A potential explanation for this may be that cancer patients believe by engaging in multiple lifestyle strategies that they will reduce their risk of recurrence and increase their sense of control, both of which may improve subjective or percieved QoL. With this in mind our population may further improve their outcomes through combining CIT with other lifestyle recommendations such as increased physical activity.

This research was designed as an exploratory study conducted over a single five day period at a series of patient support centres with participants completing a questionnaire on an isolated occasion. Therefore, there was no assessment of the impact of specific CIT or any changes from the initiation of contact with the centres. Future research should assess PROs before and after receiving support at integrative oncology centres, in addition to the assessment of the impact of individual therapies. Given the importance of the relationship between doctor and patient, and the low disclosure rates regarding CIT, future research should also incorporate further exploration of the experiences of medical professionals and the integration of supportive services within traditional medical systems. Additionally, with the majority of respondents being women, despite equally high rates of cancer in males, it is important for support services to be directed towards men and the medical professionals involved in their treatment. This should be considered in future research and service provision. Despite two members of the research team being affiliated with SolarisCare they had no direct impact with participants or significant involvement in data collection therefore presenting no potential influence of findings.

## Conclusions

The aims of this study were to determine medical and demographic characteristics of patients accessing support services at integrative oncology centres across Western Australia. Key findings from the study included: (a) a large proportion of the sample (80%) had at least one other comorbid condition, (b) QoL was significantly lower than comparative populations, (c) half of the sample were not meeting recommended physical activity guidelines, and (d) significantly poorer outcomes were reported for those in the younger age group, despite no differences for gender.

A greater understanding of the health profiles of patients presenting to supportive care centres and their use of CIT, provides health professionals with key information to ensure the safety of supportive care practices, as well as fosters optimal patient outcomes and enhances the integration of supportive care strategies within mainstream medical care.

## Abbreviations

PROs: Patient rated outcomes; CIT: Complementary and integrated therapies; QoL: Quality of life; CRF: Cancer related fatigue; FACT-G: Funcational assessment for cancer therapy – general; SF-36: Short form 36 health survey; SCFS: Schwartz cancer fatigue scale; MJM: Muscle joint measure; IPAQ: International physical activity questionnaire; PCS: Physical component score; MCS: Mental component score; GH: General health; SF: Social functioning; SWB: Social wellbeing; FWB: Functional wellbeing; PWB: Physical wellbeing.

## Competing interests

The authors declare that they have no competing interests.

## Authors’ contributions

All authors participated in the conception and design of the study, and have been involved in the drafting and revising of the manuscript for critically important intellectural content. BF, KW and AP coordinated the collection of data and analysis, with BF and KW conducted the analysis and interpretation under the supervision of the entire research team. All authors have read and approved the final manuscript.

## Pre-publication history

The pre-publication history for this paper can be accessed here:

http://www.biomedcentral.com/1472-6882/13/169/prepub

## Supplementary Material

Additional file 1 SNAPSHOT Questionnaire 1. Click here for file
