# Supplementary material for: Characteristics and quality of life of patients presenting to cancer support centres: patient rated outcomes and use of complementary therapies
Source: BMC Complement Altern Med. 2013 Jul 11;13:169. doi: 10.1186/1472-6882-13-169 (PMC3717092; doi:10.1186/1472-6882-13-169)
Supplement: Additional file 1 — SNAPSHOT Questionnaire 1. [file 1472-6882-13-169-S1.pdf]

SolarisCare
Integrated care for cancer

THE UNIVERSITY OF WESTERN AUSTRALIA
Achieve International Excellence

SNAPSHOT Questionnaire 1

Consent

By completing this questionnaire, I acknowledge I have read the information provided and any questions I have asked have been answered to my satisfaction. I **agree to participate** in this randomised trial, realising that I may withdraw at any time without reason and without prejudice.

I understand that all information provided is treated as **strictly confidential** and will not be released by the investigator unless required to by law. I have been advised as to what data are being collected, what the purpose is, and what will be done with the data upon completion of the research.

I agree that research data gathered for the study may be published provided my name or other identifying information is not used.

Signed (*Participant*) Date

Name: ____

Date of Birth: *(day)* _________**/** *(month)* _________**/ 19**_______

Gender: Male / Female

SolarisCare Centre:
☐ Sir Charles Gairdner Hospital ☐ St John of God ☐ South West - Bunbury ☐ Albany

Employment Status:
Are you currently working? *(Please circle)* YES / NO

What is your current employment status? (Select one)
☐ Casual ☐ Part Time ☐ Full Time ☐ Retired *(since diagnosis? Y / N)*

☐ Sick leave *If so, how long:* ____________________*months*

Cancer Information:
When were you first diagnosed with cancer? (Month/year) ______________________________________

Location of cancer: ____

Has your cancer spread? YES / NO *If yes, where*? ____

Treatment Received:

☐ Radiation Therapy	⇒	☐ Planned	☐ Current/Ongoing	☐ Past
☐ Chemotherapy	⇒	☐ Planned	☐ Current/Ongoing	☐ Past
☐ Cancer Surgery	⇒	☐ Planned	☐ Current/Ongoing	☐ Past
☐ Hormone Treatment	⇒	☐ Planned	☐ Current/Ongoing	☐ Past
☐ Other: ______________________________			☐ Current/Ongoing	☐ Past

SolarisCare
Integrated care for cancer

The University of Western Australia
Achieve International Excellence

Other Health Information: *(Please tick all that apply – remember any daily medications)*

- ☐ Cardiac condition (i.e. high blood pressure, heart disease)
- ☐ Circulatory Problems
- ☐ Diabetes
- ☐ Epilepsy
- ☐ Arthritis or joint problems
- ☐ Respiratory Problems (incl. Asthma)
- ☐ Osteoporosis, osteopenia or bone fracture/s
- ☐ Digestive Problems
- ☐ Thrombocytopaenia
- ☐ Neutropaenia
- ☐ Persistent or chronic pain
- ☐ Depressive or anxiety disorder
- ☐ Neurological condition (i.e. multiple sclerosis, dementia, Parkinson's, Motor Neurone Disease etc)
- ☐ Other: ____

Are you seeing any health professionals other than an Oncologist or Haematologist? *(Please tick all that apply)*

- ☐ Psychologist/Councillor
- ☐ Dietician/Nutritionist
- ☐ Physiotherapist/Chiropractor
- ☐ Naturopath/Herbalist
- ☐ Support Group/Forum
- ☐ Yoga/Pilates/Exercise Professional
- ☐ Other: ______________________________

Are you taking any supplements or natural remedies? YES / NO

If yes, are they… ☐ prescribed by a health professional? ☐ self-selected?

Please list supplements or remedies used *(i.e. Multiple vitamin, herbal tonic, vitamin injections)*

____

____

Since diagnosis how many sessions of complementary therapy have you had? *(Please tick appropriate)*

☐ Less than 6 sessions ☐ Between 6 -12 ☐ More than 12 sessions

What services or therapies have you utilised at SolarisCare? *(Please tick all that apply)*

- ☐ Acupuncture
- ☐ Aromatherapy
- ☐ Bowen Therapy
- ☐ Guided Meditation
- ☐ Counselling
- ☐ Creative Art Therapy
- ☐ Healing Touch
- ☐ Kinesiology
- ☐ Pranic Healing
- ☐ Qi Gong
- ☐ Reflexology
- ☐ Reiki
- ☐ Relaxation Massage
- ☐ Resource Library
- ☐ Waiting area/lounge
- ☐ Other: ______________________________

PLEASE CONTINUE ON NEXT PAGE…

SolarisCare
Integrated care for cancer

THE UNIVERSITY OF WESTERN AUSTRALIA
Achieve International Excellence

Schwartz Cancer Fatigue Scale

The words and phrases describe different feelings people associate with fatigue. Please read each item and **circle the number that indicates how much fatigue has made you feel in the past 2 to 3 days.**

	NOT AT ALL	A LITTLE BIT	MODERATELY	QUITE A BIT	EXTREMELY
Tired	1	2	3	4	5
Difficulty thinking	1	2	3	4	5
Overcome	1	2	3	4	5
Listless	1	2	3	4	5
Worn out	1	2	3	4	5
Helpless	1	2	3	4	5

PLEASE CONTINUE ON NEXT PAGE…

SolarisCare
Integrated care for cancer

THE UNIVERSITY OF WESTERN AUSTRALIA
Achieve International Excellence

INTERNATIONAL PHYSICAL ACTIVITY QUESTIONNAIRE

We are interested in finding out about the kinds of physical activities that people do as part of their everyday lives. The questions will ask you about the time you spent being physically active in the **last 7 days**. Please answer each question even if you do not consider yourself to be an active person. Please think about the activities you do at work, as part of your house and yard work, to get from place to place, and in your spare time for recreation, exercise or sport.

Think about all the **vigorous** activities that you did in the **last 7 days**. **Vigorous** physical activities refer to activities that take hard physical effort and make you breathe much harder than normal. Think *only* about those physical activities that you did for at least 10 minutes at a time.

1. During the **last 7 days**, on how many days did you do **vigorous** physical activities like heavy lifting, digging, aerobics, or fast bicycling?

    _____ **days per week**

    ☐ No vigorous physical activities → ***Skip to question 3***

2. How much time did you usually spend doing **vigorous** physical activities on one of those days?

    _____ **hours per day**

    _____ **minutes per day**

    ☐ Don't know/Not sure

Think about all the **moderate** activities that you did in the **last 7 days**. **Moderate** activities refer to activities that take moderate physical effort and make you breathe somewhat harder than normal. Think only about those physical activities that you did for at least 10 minutes at a time.

3. During the **last 7 days**, on how many days did you do **moderate** physical activities like carrying light loads, bicycling at a regular pace, or doubles tennis? Do not include walking.

    _____ **days per week**

    ☐ No moderate physical activities → ***Skip to question 5***

4. How much time did you usually spend doing **moderate** physical activities on one of those days?

_____ **hours per day**

_____ **minutes per day**

☐ Don't know/Not sure

Think about the time you spent **walking** in the **last 7 days**. This includes at work and at home, walking to travel from place to place, and any other walking that you might do solely for recreation, sport, exercise, or leisure.

5. During the **last 7 days**, on how many days did you **walk** for at least 10 minutes at a time?

_____ **days per week**

☐ No walking → ***Skip to question 7***

6. How much time did you usually spend **walking** on one of those days?

_____ **hours per day**

_____ **minutes per day**

☐ Don't know/Not sure

The last question is about the time you spent **sitting** on weekdays during the **last 7 days**. Include time spent at work, at home, while doing course work and during leisure time. This may include time spent sitting at a desk, visiting friends, reading, or sitting or lying down to watch television.

7. During the **last 7 days**, how much time did you spend **sitting** on a **week day**?

_____ **hours per day**

_____ **minutes per day**

☐ Don't know/Not sure

This is the end of the questionnaire bundle one,

THANK YOU **for participating!!**
